# Ultrasound-Guided Subclavian Vein Cannulation in Neonate via Supraclavicular Approach

**DOI:** 10.1155/2017/9789427

**Published:** 2017-07-09

**Authors:** Onur Balaban, Tayfun Aydın

**Affiliations:** Department of Anesthesiology & Pain Medicine, Dumlupinar University Hospital, Merkez, Kutahya, Turkey

## Abstract

Central venous cannulation of infants may be challenging. Ultrasonography is recommended and has been found superior to classic landmark technique in pediatric central venous cannulation. The cannulation of the subclavian vein using supraclavicular approach under real-time ultrasound guidance is a novel technique. It may have advantages over ultrasound-guided jugular vein cannulation in specific patients. We report a case of 3200-gram 20-day-old anencephalic neonate who had a diffuse generalized edema. The neonate was cannulated successfully via subclavian vein using supraclavicular approach under ultrasound guidance.

## 1. Introduction

Central venous cannulation in infants weighing less than 10 kilograms or younger than 1 year may be difficult even with ultrasound (US) guidance. Younger age decreases success rate and increases complication rate of central venous cannulation. Ultrasonography is recommended and has been found superior to classic landmark technique, especially for pediatric patients [1, 2]. The Agency for Healthcare Research and Quality reported that the use of US guidance for the placement of central venous catheters is one of the highest patient safety practices with the strongest evidence [3]. The use of US during catheterization in pediatric patients is also recommended by the National Institute of Clinical Excellence (NICE) [4]. In pediatric patients, ultrasound-guided central venous catheter placement decreases complications and decreases placement attempts compared with the landmark technique [5].

There are many approaches and techniques using ultrasound guidance during cannulation. The cannulation of the subclavian vein (SCV) using supraclavicular approach under real-time US guidance is a novel technique. It may have advantages over ultrasound-guided jugular vein cannulation in specific patients. We report a case of US-guided subclavian vein cannulation in a neonate who had a short neck and diffuse edema, using supraclavicular approach.

## 2. Case

**Figure 1:**
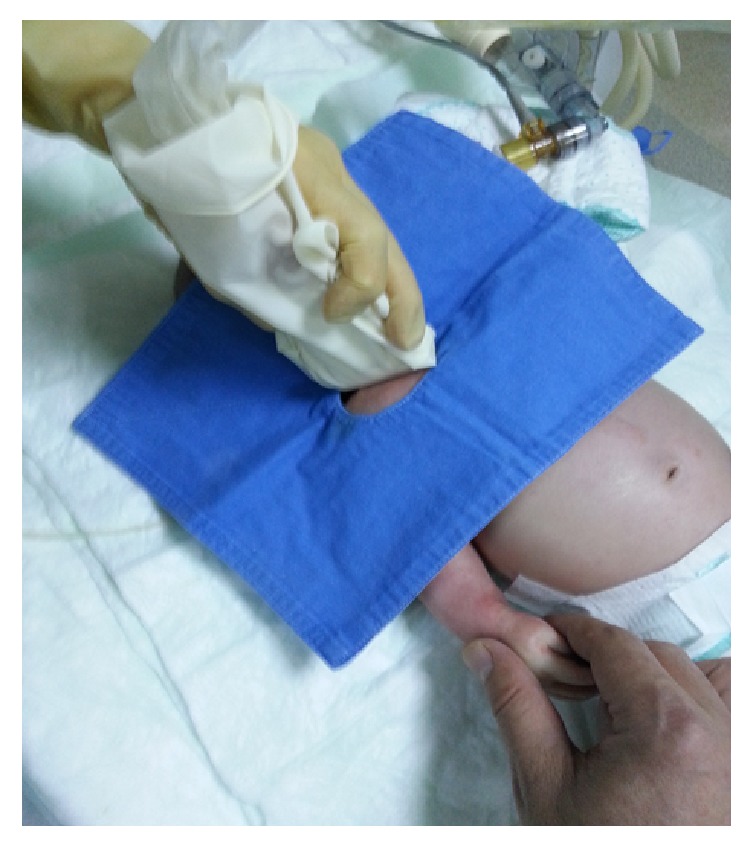
The position of the patient and ultrasound transducer during cannulation.

**Figure 2:**
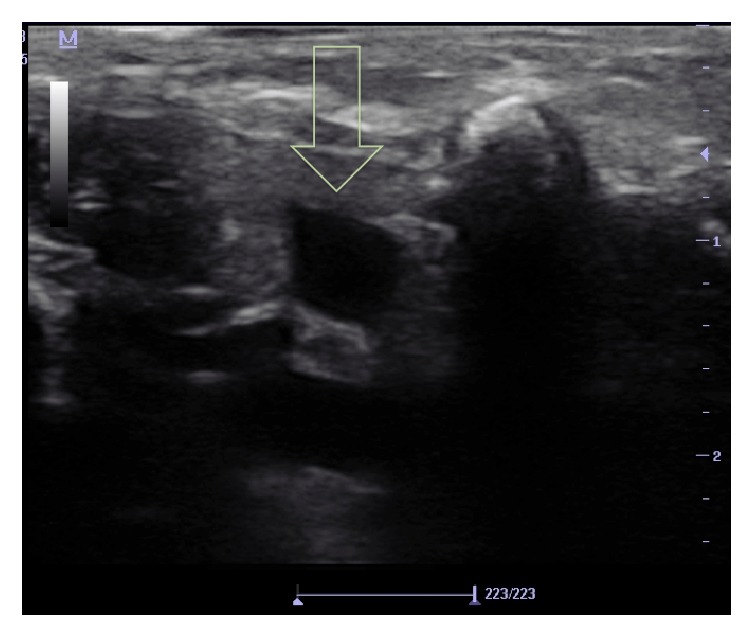
The ultrasound image of the subclavian vein. Yellow arrow is showing the subclavian vein.

Informed consent was obtained from the family of the patient for the case report. A 3200-gram, 20-day-old neonate was intubated after delivery and being followed in the neonatal intensive care unit. Multiple peripheral vascular access attempts were unsuccessful. The neck was short and there was a generalized edema involving the neck. As there was not enough space for needle insertion and manipulation at the neck for jugular cannulation, we decided to place a subclavian vein catheter under US guidance using supraclavicular approach. An experienced anesthetist about US-guided jugular and subclavian vein cannulation in adults and infants performed the cannulation. Before the procedure, 5 milligrams per kilogram intramuscular ketamine was administered. The neonate was placed in supine position with the head turned to the opposite of needle insertion site (Figure 1). A sheet was placed under the infant's shoulder for an optimum position to facilitate US scanning and cannulation. The anesthetist stood at the right side of the infant and performed the cannulation. A nurse pulled the right arm of the infant along the body during the procedure. The skin was prepared for aseptic condition and a 10-megahertz frequency linear transducer was placed in a sterile cover. Sterile gel was used as coupling agent. The jugular vein and carotid artery were visualized transversally at the tracheal cartilage level. Then the transducer was moved laterally and rotated slightly, following the jugular vein, until SCV was visualized. A transverse cross-sectional image of SCV was visualized at supraclavicular region. Also, subclavian artery was visualized posterior to the vein (Figure 2). There was no enough space for alignment and tilting maneuvers of the transducer due to anatomical difficulties of the patient and pleura could not be visualized. Vascular flux was verified by color Doppler before the puncture to identify subclavian artery and the SCV. Using out-of-plane technique and visualizing the transverse cross-sectional view of SCV, the needle was inserted into the vein visualizing the tip under real-time US guidance. An open-end needle was inserted without an attached syringe, to avoid collapse of the vein when negative pressure is applied by syringe. When the tip of the needle was seen in the vein and blood reflux was observed, the guide wire was inserted through the needle into the vein. Then, a 22-gauge catheter (Certofix Mono Paed S 110, Braun, Melsungen, Germany) was introduced over the guide wire. The procedure was completed at first attempt, without any complication such as hematoma, arterial puncture, or pneumothorax. The place of catheter in SCV was verified by US scan and a chest X-ray.

## 3. Discussion

Ultrasound guidance technique is becoming gold standard for central venous cannulation. The success rate is increased and the complications related to central venous catheter (CVC) placement are decreased [6]. Failure rates for CVC placement in children range from 5% to 19% with reported complication rates from 2.5% to 22% [5]. US-guided CVC placement decreases complications and increases the success rate at first attempt compared to the landmark technique in pediatric patients [5]. A meta-analysis by Hind et al. reported that the use of 2-dimensional US guidance was associated with increased success of cannulation of internal jugular vein and SCV [1]. Another meta-analysis of 26 randomized controlled trials showed that patients receiving CVC can obtain significant benefit from US [7].

There are many techniques and approaches for central venous cannulation. Internal jugular, subclavian, and femoral vein are commonly used sites [1]. The femoral vein is the most common attempted site during pediatric resuscitation in emergency departments [2]. Femoral vein cannulation is associated with a low complication rate during insertion in hypovolemic patients or patients with low cardiac output, compared to the internal jugular and subclavian catheters [8]. Disadvantages of femoral cannulation are a higher incidence of thromboembolic and infectious complications [2]. Although the most popular site has been the femoral vein, recently, the internal jugular vein (IJV) has been favored when ultrasound is used [6]. During cannulation, IJV may collapse under probe or needle pressure. High puncture of the IJV often is exposed to difficulties like tunnelization difficulty because of the neck shortness, whereas low puncture of the IJV hampers the insertion of the metallic guide that gets stopped against the medial wall [9]. Subclavian vein cannulation does not present these difficulties because of its anterior wall fixation on clavicle. This brings protection against venous collapse and enables lateral insertion of needle, allowing an axial insertion of the metallic guide [9]. SCV is less affected by collapse and easily accessed to tunnel and fixed to a patient's shoulder [10]. Infraclavicular placement of the CVC is not recommended for a long period of time, because of the risk of catheter rupture caused by the costoclavicular pinch [9]. Supraclavicular cannulation of SCV may be more suitable for long-term cannulation.

Reports on pediatric SCV cannulation have been rare. A supraclavicular approach was suggested by Yoffa and known since 1965 but was rarely used [11]. Physicians have been hesitant to use this technique in blind puncture, because of the pleural and vascular closeness of insertion site. In a retrospective analysis, the authors used subclavian approach as first choice and cannulated 148 pediatric patients [12]. They concluded that subclavian central venous cannulation is a safe procedure with minimal complications in pediatric patients. Pirotte and Veyckemans described a novel US-guided approach for SCV cannulation in infants and children [13]. The principle of this technique is to place the US probe at the supraclavicular level to obtain a longitudinal view of the SCV. This approach offers a longitudinal view of intrathoracic segment of the SCV reaching (via the innominate vein on left side) the IJV at the level of the Pirogoff confluent. The authors reported a high success rate and no major complications. The limitation of this approach is that the US shadow of the clavicle may not allow following the entire course of the needle. Guilbert et al. placed CVCs to the SCV of 40 children and neonates under US guidance [10]. They scanned the internal jugular vein from thyroid cartilage level to the supraclavicular region until the brachiocephalic vein and the SCV could be viewed in long axis. Viewing the brachiocephalic vein and the SCV in long axis, they performed needle in-plane puncture under ultrasound guidance. Rhondali et al. used a supraclavicular technique for SCV cannulation in 37 infants [9]. They placed the US probe at the supraclavicular level to obtain a longitudinal view of the SCV and gain access to the vein by in-plane needle puncture. Park et al. reported a case series of SCV cannulation in 11 pediatric patients weighing 1.1 kg to 15 kg [6]. They visualized the vein using infraclavicular approach and performed the puncture using needle in-plane technique. Kulkarni et al. reported cannulation of subclavian vein by a supraclavicular approach under ultrasound guidance in a series of 150 children and they routinely used this method in pediatric cardiac surgery patients [14]. Nardi et al. used an in-plane technique and a longitudinal axis visualization of subclavian vein in a prospective observational study for pediatric patients [15]. Ultrasound-guided subclavian vein cannulation was found feasible in low birth weight even weighing less than 1,500 g [16].

All of these studies confirm that US helps to find the location of the vascular structures, demonstrates preexisting intravascular clots, and defines anatomically inaccessible vessels. As a different approach from these studies, we preferred needle out-of-plane technique visualizing a transverse cross-sectional view of the subclavian vein. The patient's neck anatomy and diffuse generalized edema did not allow a perfect longitudinal view of the SCV. This approach provided the optimal ultrasonographic vision of the vein at the supraclavicular region.

In conclusion, needle out-of-plane technique with visualization of transverse cross-sectional view of subclavian vein is a successful alternative for supraclavicular subclavian vein cannulation in specific patients. Our case supports recent previous studies that are showing positive results for ultrasound-guided supraclavicular puncture of the subclavian vein in pediatric patients.
